# Gurvits syndrome: a case of acute esophageal necrosis associated with diabetic ketoacidosis

**DOI:** 10.1186/s12876-022-02349-z

**Published:** 2022-06-02

**Authors:** Daisuke Kitawaki, Atsushi Nishida, Keitaro Sakai, Yuji Owaki, Kyohei Nishino, Yoshika Noda, Hirotsugu Imaeda

**Affiliations:** Department of Gastroenterology and Hepatology, Nagahama City Hospital, 313 Oinuicho, Nagahama, 526-8580 Shiga Japan

**Keywords:** Acute esophageal necrosis, Gurvits syndrome, Black esophagus, Acute necrotizing esophagitis, Diabetic ketoacidosis, Case report

## Abstract

**Background:**

Acute esophageal necrosis (AEN), commonly referred to as Gurvits syndrome or “black esophagus”, is a rare clinical disease. We present a case of AEN associated with diabetic ketoacidosis (DKA).

**Case presentation:**

A 66-year-old male came to our hospital with coffee-ground emesis, dyspnea, and general malaise. He was treated for type 2 diabetes mellitus using insulin and had not been taking his medication, including insulin, for several days. Laboratory analysis revealed severe hyperglycemia (730 mg/dL), normocytic anemia (hemoglobin level, 7.7 g/dL; mean corpuscular volume, 100.4 fL), high serum potassium (7.6 mEq/L), and a high level of blood urea (98.7 mg/dL). Ketones and glucose were detected in the urine, and serum β-hydroxybutyrate was elevated (2132 µmol/L). Arterial blood gas analysis confirmed metabolic acidosis (pH, 7.29; HCO_3_, 10.5 mmol/L). Collectively, the patient was diagnosed with DKA and upper gastrointestinal bleeding. The patient’s condition improved with intravenous fluids, and he received intravenous insulin to treat DKA. According to these findings, the patient was diagnosed with DKA and upper gastrointestinal bleeding. The patient underwent esophagogastroduodenoscopy (EGD) which revealed a circumferential necrosis of the middle and distal esophagus, immediately proximal to the gastroesophageal junction. The patient was then treated with an intravenous proton pump inhibitor. The patient continued to improve with conservative treatment and was subsequently discharged in a stable condition. An EGD repeated 14 days after discharge showed complete healing of the necrotic-like mucosal change without stricture formation of the esophagus.

**Conclusions:**

AEN is rare but potentially life-threatening case of upper gastrointestinal bleeding. Therefore, a clinician should be aware of AEN as a potential cause of upper gastrointestinal bleeding in elderly patients with poorly controlled diabetes and significant comorbidities.

## Background

Acute esophageal necrosis (AEN), commonly referred to as Gurvits syndrome, “black esophagus”, or “acute necrotizing esophagitis”, is a rare cause of upper gastrointestinal bleeding. It was first described in the medical literature in 1990 by Goldenberg et al., and was came into the limelight by Gurvits et al. in 2007 [1, 2]. In endoscopic examinations, the prevalence of AEN is rare and reported to be up to 0.28% [3, 4]. AEN is markedly skewed by gender and age. Men are affected four times as often as women, and although AEN can present at all ages, the peak age of incidence is the sixth decade of life with an average age of 67 years [3]. The precise etiology of the disease remains unknown, but is presumed to be multifactorial, namely ischemia of mucosa, exposure of the esophageal mucosa to chemical contents in the stomach, and deterioration of mucosal barrier associated with poor physical condition [3, 4]. It was reported that most of the older patients had multiple comorbidities, including diabetes mellitus, coronary artery disease, renal insufficiency, alcohol abuse, and hypertension [5]. Significant vascular damage associated with diabetes mellitus can be an important contributor to the development of esophageal necrosis [3, 6–8].

Clinical manifestations are marked by upper gastrointestinal bleeding, with most patients presenting with hematemesis or melena [3, 4]. Serious acute complications include perforation and mediastinitis [9]. The most frequent long-term complication is stenosis formation, which is reported to occur in about 10% of cases [3, 9]. A mortality rate as high as 32% has been reported, likely related to comorbidities [3, 4]. The mortality rate associated with AEN is approximately 6%, primarily due to esophageal perforation [2–4, 9]. Although a standard treatment for AEN is not yet established, most reports have recommended the treatment of coexisting clinical conditions, systemic resuscitation with intravenous fluid therapy, glycemic control, use of aggressive intravenous proton pump inhibitors, and parenteral nutrition [3, 4, 9].

Here, we report a case of Gurvits syndrome induced by diabetic ketoacidosis (DKA).

## Case presentation

A 66-year-old male came to our hospital with coffee-ground emesis, dyspnea, and general malaise. He also had abdominal pain and nausea. His medical history included type 2 diabetes mellitus, obstructive pulmonary disease, and alcohol abuse. He had not been taking his medication, including insulin, for several days due to nausea. His vital signs were as follows: Glasgow Coma Scale, 15 (E4V5M6); body temperature, 35.5 ℃; blood pressure, 103/79 mmHg; pulse, 100 beats per minute; and percutaneous oxygen saturation with a flow rate of 2 L per minute through a nasal cannula, 100%. A physical examination revealed pallor conjunctiva and a coffee powder-like substance around the mouth. The abdomen was mildly tender with no muscular defense. Other physical examinations revealed no remarkable findings. Laboratory analysis revealed severe hyperglycemia (730 mg/dL), normocytic anemia (hemoglobin level, 7.7 g/dL; mean corpuscular volume, 100.4 fL), high serum potassium (7.6 mEq/L), and a high level of blood urea (98.7 mg/dL). Ketones and glucose were detected in the urine, and serum β-hydroxybutyrate was elevated (2132 µmol/L). Arterial blood gas analysis confirmed metabolic acidosis (pH, 7.29; HCO_3_, 10.5 mmol/L) (Table 1). Collectively, the patient was diagnosed with DKA and upper gastrointestinal bleeding.

**Table 1:** Laboratory data on admission

*Peripheral blood tests*			ALT	122	IU/L
WBC	11	×103/µL	CPK	141	IU/L
Neu	74.2	%	LDH	224	IU/L
Ly	16.9	%	ALP	54	U/L
Eo	0	%	γ-GTP	52	IU/L
RBC	2.27	×106/µL	Glucose	730	mg/dL
Hb	7.7	g/dL	HbA1c (NGSP)	8	%
Ht	22.8	%	CRP	6.03	mg/dL
MCV	100.4	fL	eGFR	27.5	mL/min/1.73 m^2^
MCH	33.9	pg	BNP	61.8	pg/mL
MCHC	33.8	%	Cortisol	27.5	mg/dL
Platelet	266	×103/µL	β-hydroxybutyrate	2132	µmol/L
*Coagulation study*			*Arterial blood gas analysis* (*O*_*22L/min cannula*_)		
PT	13.6	s	pH	7.294	
APTT	26.2	s	PCO_2_	21.9	mmHg
PT-INR	1.06		PO_2_	140.2	mmHg
D-dimer	4.33	µg/mL	HCO_3_	10.5	mmol/L
*Biochemistry tests*			BE	− 14.6	mmol/L
TP	4.9	g/dL	Lactate	12.09	mmol/L
Alb	2.9	g/dL	*Urinalysis*		
BUN	98.7	mg/dL	pH	≦ 5.0	
Cr	1.99	mg/dL	Protein	–	
UA	12.3	mg/dL	Glucose	4+	
Na	120	mEq/L	Ketone	1+	
K	7.6	mEq/L			
Cl	82	mEq/L	*Others*		
Ca	8.6	mg/dL	Plasma osmolality	334	mOsm/L
T-Bil	0.53	mg/dL	Urine osmolality	501	mOsm/L
AST	54	IU/L			

*WBC* white blood cell, *Neu* neutrophils, *Ly* lymphocytes, *Eo* eosinophils, *RBC* red blood cell, *Hb* hemoglobin, *Ht* hematocrit, *MCV* mean corpuscular volume, *MCH* mean corpuscular hemoglobin, *MCHC* mean corpuscular hemoglobin concentration, *PT* prothrombin time, *APTT* activated partial thromboplastin time, *PT-INR* prothrombin time-international normalized ratio, *TP* total protein, *Alb* albumin, *BUN* blood urea nitrogen, *Cr* creatinine, *UA* uric acid, *Na* sodium, *K* potassium, *Cl* chlorine, *Ca* calcium, *T-Bil* total-bilirubin, *AST* aspartate aminotransferase, *ALT* alanine aminotransferase, *CPK* creatinine phosphokinase, *LDH* lactate dehydrogenase, *ALP* alkaline phosphatase, *γ-GTP* γ-glutamyl transpeptidase, *NGSP* National Glycohemoglobin Standardization Program, *CRP* C-reactive protein, *eGFR* estimated glomerular filtration rate, *BNP* brain natriuretic peptide, *BE* base excess

**Fig. 1:**
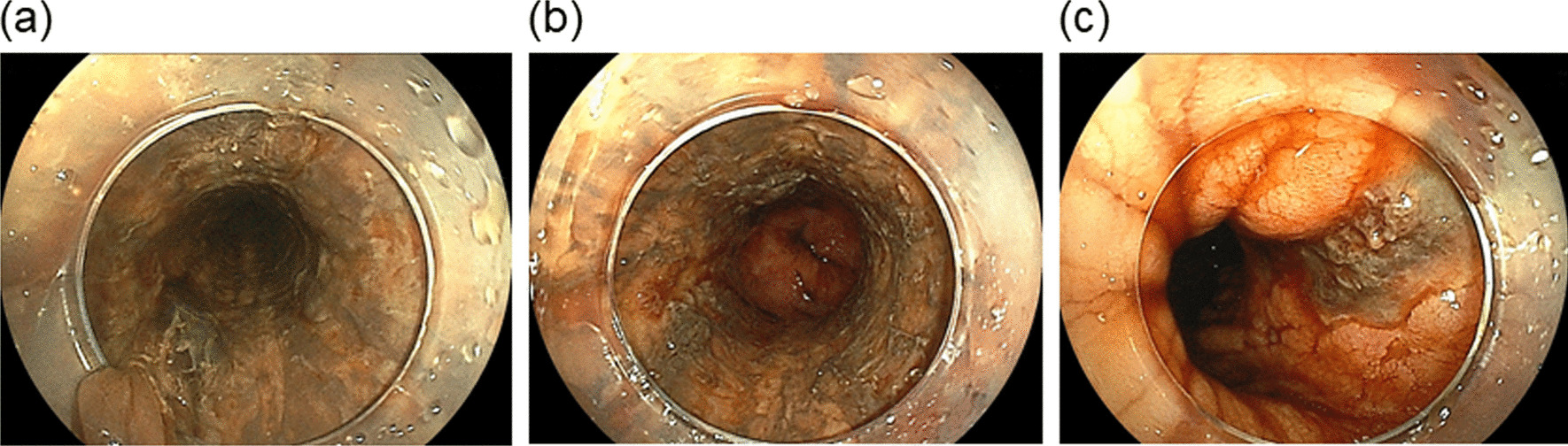
Esophagogastroduodenoscopy on admission showed a circumferential, diffusely necrotic mucosa necrosis from the middle **a** to distal portions, **b** of the esophagus with an abrupt transition at the gastroesophageal junction. **c** Gastric ulcer on the lesser curvature of the upper body of the stomach with an exposed blood vessel

**Fig. 2:**
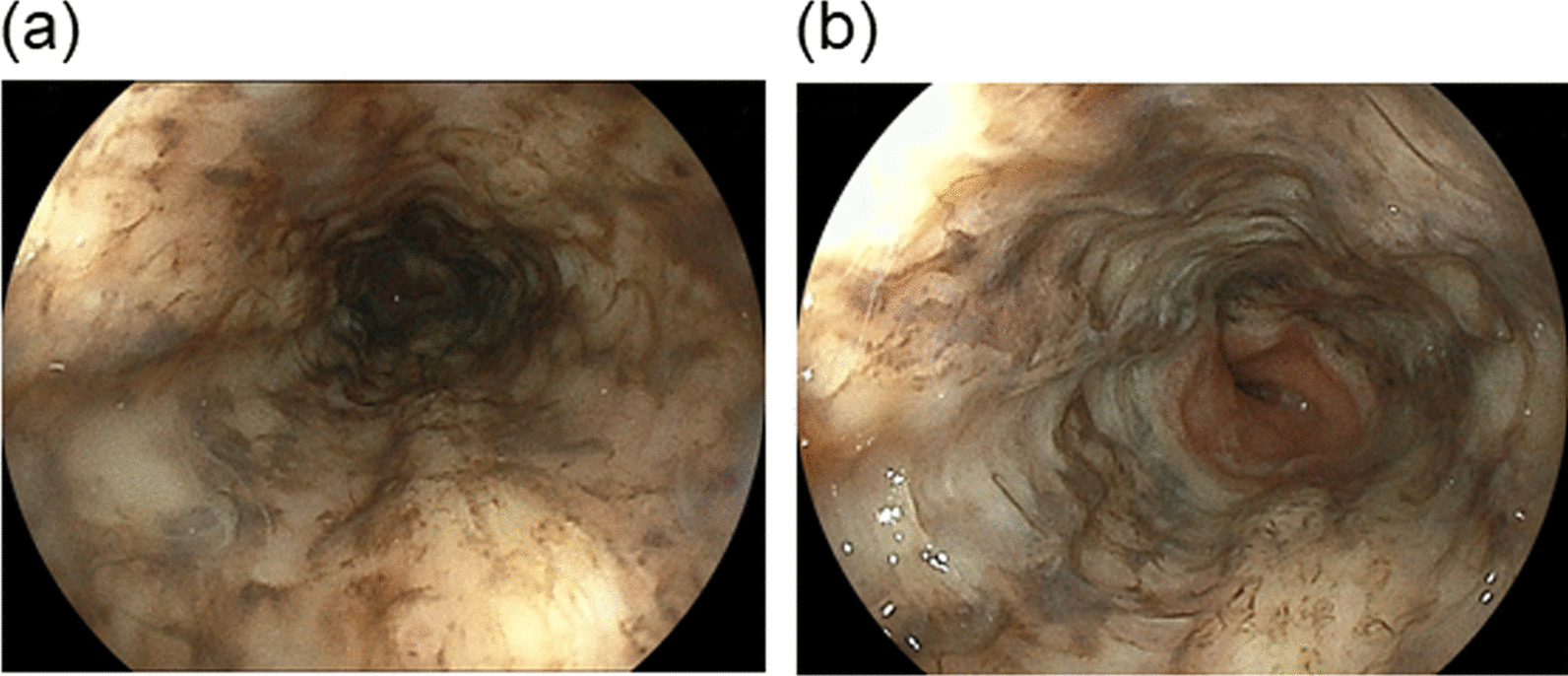
Images from a second esophagogastroduodenoscopy performed one day after admission. A circumferential black discoloration of mucosa from the middle (**a**) to the distal portion of the esophagus (**b**)

**Fig. 3:**
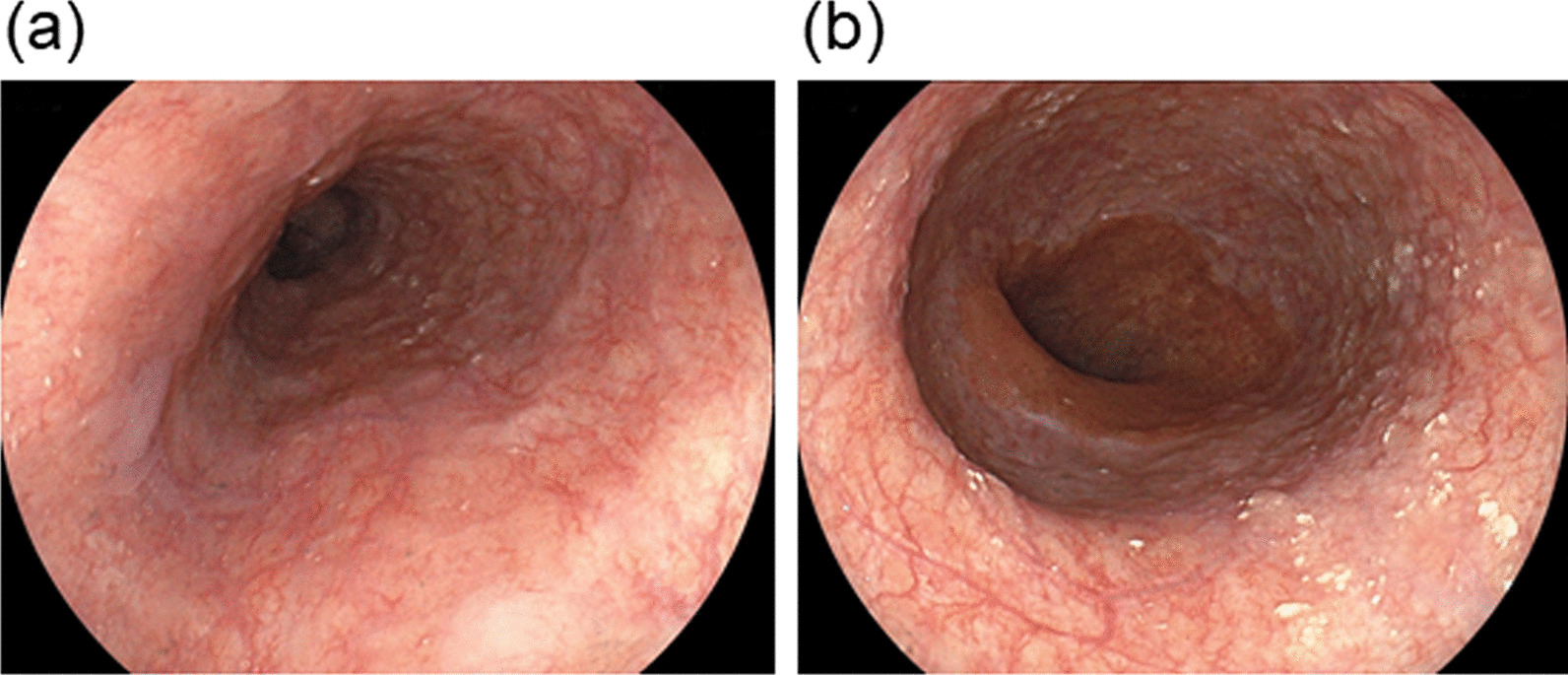
Esophagogastroduodenoscopy performed 14 days after discharge. A complete healing of the necrotic-appearing mucosal changes from the middle (**a**) to the distal portion (**b**) of the esophagus

The patient improved with intravenous fluids, and was given insulin intravenously to treat DKA. After about 48 h of insulin administration, the daily plasma glucose profile improved. The patient underwent esophagogastroduodenoscopy (EGD) which revealed a circumferential necrosis of the middle and distal esophagus, immediately proximal to the gastroesophageal junction (Fig. 1a, b). At computed tomography showed thickening of the distal esophagus, but didn’t detect the presence of esophageal perforation. EGD also revealed a gastric ulcer on the lesser curvature of the upper body of the stomach with an exposed blood vessel (Fig. 1c). The exposed blood vessel was cauterized with hemostatic forceps. From the endoscopic findings, acute necrotizing esophagitis and hemorrhagic gastric ulcer were diagnosed. The patient was then treated with an infusion proton pump inhibitor intravenously. A second EGD, performed one day after admission, showed no remarkable findings with circumferential black discoloration at the middle and distal portion of esophagus as compared to those the day before (Fig. 2a, b), and hemostasis of the gastric ulcer. The patient continued to improve with conservative treatment and was subsequently discharged in a stable condition. An EGD repeated 14 days after discharge showed complete healing of the necrotic-like mucosal change without esophageal stricture formation (Fig. 3a, b).

## Discussion and conclusion

AEN, also known as Black esophagus or Gurvits syndrome, was first described by Goldenberg et al. in 1990 [1], and came to prominence after a series of cases were reported in 2007 [2]. AEN is a rare disease with an estimated prevalence of up to 0.28% [3, 4]. Endoscopic findings are characterized by the development of diffuse marginal black mucosa in the distal esophagus, with a sudden transition to normal mucosa at the gastroesophageal junction [1–4, 7, 10].

Though the exact cause of the disease is unknown, it is presumed to be a multifactorial disease in which ischemia, impaired intrinsic mucosal barriers, and massive regurgitation of gastric contents that acutely exceeds the protective and regenerative capacity of vulnerable esophageal mucosa causing both ischemic and chemical injury of the esophagus [3, 4, 8, 11–13]. The blood supply of the esophagus is segmental. The branches of inferior thyroid artery supply the upper esophagus. The middle esophagus receives its arterial supply from vascular branches from aorta and intercostals. The distal esophagus, which has the fewest vessels, is supplied from the esophageal branches of left gastric artery. Because of its low blood supply, the distal esophagus is more susceptible to ischemic injury and the necrotic change is most pronounced in this region [2, 14, 15]. Moreover, in our case, EGD also revealed a gastric ulcer an exposed blood vessel. Therefore, acute blood loss caused by a hemorrhagic gastric ulcer may contribute to ischemia of esophagus, leading to the development of AEN. Chronic predisposing conditions and malnutrition may also contribute to decreased the buffering capacity of esophageal mucosa and may enhance mechanisms by which esophageal necrosis is progressed.

The diagnosis of AEN or Gurvits syndrome is based on endoscopic findings consisting a circumferentially black esophageal mucosa that abruptly terminates at the gastroesophageal junction [1–4, 7, 10]. AEN affects predominately elder male with a number of medical comorbidities [2–4]. Demographically, almost 90% of patients with AEN are suffered from hyperglycemia [16, 17]. Moreover, disease associations include vascular disorders, hypertension, chronic kidney disease, cancer, malnutrition, gastric outlet obstruction, and in this case, diabetes mellitus and alcohol abuse [3, 4]. The clinical presentation is marked by upper gastrointestinal bleeding, with most patients presenting with hematemesis or melena, comparable to our case [3, 4]. Other gastrointestinal symptoms such as epigastric pain, chest pain, nausea, vomiting, and dysphagia may occur. Other symptoms such as fever, hypotension and syncope are less common [3, 4]. The patient showed abdominal pain and nausea. Laboratory findings are nonspecific, but associated laboratory abnormalities such as anemia, leukocytosis, and hyperlactatemia have been reported [3, 4], all of which are comparable to the present patient.

There is no established therapy for AEN. Management of AEN should be directed at correcting the underlying disease and hemodynamic resuscitation, total parenteral nutrition, and the protection with antacid therapy with aggressive intravenous proton pump inhibitors [3, 4]. Enteral nutrition is not recommended because of the risk of perforation by feeding tube unless there is persistent vomiting [3, 4, 9]. Moreover, prophylactic administration of antibiotics is not warranted unless the patient shows clinical and objective findings of serious infection [3, 4, 9, 18]. The patient presented no sign of infection. The most common long-term complication of AEN is stenosis, which can be found in about 10% of all cases [3, 4, 9]. No stricture formation was shown in our case on follow-up EGD.

The correlation between DKA and AEN has been described in the literature [16, 19–25]. DKA has been reported to be one of the most common triggers for AEN [3, 4, 9]. Although the exact mechanism remains unclear, several etiologies have been proposed. The patients with long-standing diabetes mellitus are predisposed to the development of atherosclerosis leading to an increased risk of ischemia [3, 4, 16, 21, 26, 27]. It has been suggested that malnutrition with hemodynamic instability, and hyperglycemia in DKA can lead to low vascular flow and an impaired mucosal barrier from corrosive injury of gastric contents [3, 4, 8, 16, 21, 26, 27]. Moreover, gastric fluid stagnation, can induce gastroesophageal reflux by which esophageal necrosis is accelerated [3, 4, 21]. DKA can also cause osmotic diuresis and fluid loss leading to hypoperfusion of the middle and lower thirds of esophagus [3, 4, 16, 20].

In summary, we presented a case of AEN complicating diabetic ketoacidosis. AEN remains a rare but potentially life-threatening cause of upper gastrointestinal bleeding. It is noteworthy that up to 9% of DKA patients have findings of upper gastrointestinal bleeding, but only a small number of those undergo endoscopy to identify the underlying cause [10]. Therefore, clinicians should be aware of AEN as a potential cause of upper gastrointestinal bleeding when encountering an elderly, possibly poorly controlled diabetic patient or when additional significant comorbidities are present.

## Data Availability

The images and electric medical records are available from the corresponding author on reasonable request, but the data will not be shared to protect the patient’s confidentiality.

## Abbreviations

AEN: Acute esophageal necrosis
DKA: Diabetic ketoacidosis
EGD: Esophagogastroduodenoscopy
